# A Linked Community and Health Facility Intervention to Improve Newborn Health in Cambodia: The NICCI Stepped-Wedge Cluster-Randomized Controlled Trial

**DOI:** 10.3390/ijerph17051559

**Published:** 2020-02-28

**Authors:** Chivorn Var, Richard A. Oberhelman, Tian Shu, Supheap Leang, Ryan Duggal, Jennifer Le, Alessandra N. Bazzano

**Affiliations:** 1 National Institute of Public Health, #2 Kim Y Sung Blvd, Tuol Kork, P.O. Box 1300, Phnom Penh 12150, Cambodia; chivorn@rhac.org.kh (C.V.); leangsupheap@yahoo.com (S.L.); 2 Reproductive Health Association of Cambodia (RHAC), #5, 150 St., Phnom Penh 12150, Cambodia; 3 Department of Global Community Health and Behavioral Sciences, Tulane University School of Public Health and Tropical Medicine, 1440 Canal St., New Orleans, LA 70112, USA; oberhel@tulane.edu; 4 Department of Epidemiology, Tulane University School of Public Health and Tropical Medicine, 1440 Canal St., New Orleans, LA 70112, USA; tshu1@tulane.edu; 5 Tulane University School of Medicine, Tulane Ave, New Orleans, LA 70112, USA; rduggal@tulane.edu; 6 Louisiana Department of Health, Bureau of Family Health, New Orleans, LA 70112, USA; jle3@tulane.edu

**Keywords:** infant, newborn, Cambodia, behavior change, neonatal, community health worker, health facility

## Abstract

Background: Newborn mortality in Cambodia remains high, with sepsis and complications of delayed care-seeking important contributing factors. Intervention study objectives were to improve infection control behavior by staff in health centers; improve referral of sick newborns; increase recognition of danger signs, and prompt care-seeking at an appropriate health facility; and appropriate referral for sick newborns by mothers and families of newborn infants. Methods: The stepped-wedge cluster-randomized controlled trial took place in rural Cambodia from February 2015 to November 2016. Sixteen clusters consisted of public health center catchment areas serving the community. The intervention included health center staff training and home visits to mothers by community health volunteers within 24 h of birth and on days 3 and 7 after delivery, including assessment of newborns for danger signs and counselling mothers. The trial participants included women who had recently delivered a newborn who were visited in their homes in the first week, as well as health center staff and community volunteers who were trained in newborn care. Women in their last trimester of pregnancy greater than 18 years of age were recruited and were blinded to their group assignment. Mothers and caregivers (2494) received counseling on handwashing practices, breastfeeding, newborn danger signs, and prompt, appropriate referral to facilities. Results: Health center staff in the intervention group had increased likelihood of hand washing at recommended key moments when compared with the control group, increased knowledge of danger signs, and higher recall of at least three hygiene messages. Of mother/caregiver participants at 14 days after delivery, women in the intervention group were much more likely to know at least three danger signs and to have received messages on care-seeking compared with controls. Conclusions: The intervention improved factors understood to be associated with newborn survival and health. Well-designed training, followed by regular supervision, enhanced the knowledge and self-reported behavior of health staff and health volunteers, as well as mothers’ own knowledge of newborn danger signs. However, further improvement in newborn care, including care-seeking for illness and handwashing among mothers and families, will require additional involvement from broader stakeholders in the community.

## 1. Background

Newborn mortality remains an important public health problem in low-income countries [1,2]. It is estimated that up to 40% of under five-year-old mortality globally occurs in the newborn period [3], and among the main causes of newborn mortality are infections, especially sepsis and pneumonia [4]. In Cambodia, average newborn mortality across the country is estimated to be very high, at 19 deaths per 1000 live births, far above rates for other low middle income countries, as defined by the World Bank [5]. Interventions to reduce newborn mortality in Cambodia and many other countries have focused on improving treatment for asphyxia, particularly using the Helping Babies Breathe model [6], but the burden of possible severe bacterial infection (PSBI) is also high, and strategies to implement timely diagnosis and treatment are still urgently needed to reduce newborn mortality [7]. A verbal autopsy study in Svay Rieng, Cambodia in 2009 showed that bacterial sepsis was the likely cause in 40% of newborn deaths [8]. The Ministry of Health has up-to-date Infection Control Guidelines for health personnel, but basic infection prevention measures, such as handwashing and clean birth environment, are lacking [9]. Cambodia made strong progress in reducing maternal and under-five mortality between 2005 and 2010, but the newborn mortality rate remained unchanged during the same period [10]. The Cambodian Ministry of Health then started to develop various strategies and plans to tackle newborn mortality [11,12,13].

Among studies looking at improvement of newborn health in developing countries, many are focused on interventions conducted at community level, following success of an early study from India showing a 49.6% reduction in incidence of newborn morbidity [14]. Similarly, a community-based intervention in Bangladesh focusing primarily on infection prevention and management reduced neonatal mortality by more than 45% [15]. Another trial of a package of community-based maternal and newborn interventions in Bangladesh showed improvement in care practices and knowledge but lacked evidence of impact on mortality [16]. The study suggested taking into consideration the importance of level and cause structure of neonatal mortality in the local population in the design of the intervention. The Uganda Newborn Study (UNEST) [17], a cluster-randomized controlled trial, evaluated a home visit package intervention with health facility strengthening. The intervention significantly improved essential newborn care practices, such as immediate breastfeeding, skin-to-skin care, delayed bathing the newborn, drying umbilical cord care, but showed no difference in care-seeking for newborn illness. Economic analysis of community-based maternal and newborn care interventions [18,19,20] show a range of cost effectiveness from low (affordable for resource constrained settings) to high (unaffordable for resource constrained settings), and the World Health Organization has promoted home visiting as an important strategy for newborn health and survival [21].

We present findings from the first community-based newborn survival intervention in Cambodia, the Newborn Infection Control and Care Initiative for health facilities to accelerate reduction of newborn mortality (NICCI) [9,22]. The study was carried out in Takeo, one of 25 provinces in Cambodia, located in the southern part of the country by the Vietnamese border, with a population of 923,373. The province is subdivided into 10 administrative districts, 5 operational districts (OD), 100 communes, and 1117 villages. In Takeo, there is one provincial hospital, five operational districts with three referral hospitals, and 73 health centers. NICCI study areas included 16 health centers (HCs) across all the five operational districts and 267 villages with two village health support group members (VHSGs) per village.

## 2. Methods 

### 2.1. Objectives 

The study had three specific objectives: (1) to improve infection control behavior by staff in health centers through training on infection prevention; (2) to improve recognition and referral of sick newborns through village health worker visits; and (3) to improve knowledge of newborn danger signs and appropriate referral for sick newborns by mothers and families of newborn infants.

The study was intended to test the hypothesis that delivering a coordinated intervention combining improved education for health center midwives, village health care workers, and mothers of newborns, along with improved care coordination with an increase in the number of interactions (points of contact) between mothers and health care personnel, would be associated with improved knowledge of newborn danger signs among mothers and health care workers, more rapid case detection of significant newborn illnesses, and improved appropriate referral of ill newborns. An initial exploratory hypothesis was to assess whether the intervention reduced newborn mortality, but subsequently this outcome was removed due to insufficient power to detect mortality in the sample size.

### 2.2. Trial Design

A cluster-randomized stepped wedge trial design was utilized to assess a newborn survival intervention taking place at both health facility (cluster) and community (household) levels, involving care coordination and counseling. A stepped wedge design was used so that all communities and facilities would receive the intervention. The intervention was implemented through 16 public health centers (HCs) from February 2015 to November 2016 for births taking place at HC locations [22]. Existing community health workers, known as village health support group (VHSG) volunteers, HC midwifery staff, and study staff comprised the implementation teams. The trial protocol was approved by the National Ethics Committee Health Research of the Cambodia Ministry of Health and by the Institutional Review Boards of Tulane University. This study is registered with ClinicalTrials.gov, number NCT02271737.

The intervention was conducted at three levels: health center, community health worker, and at-home caregiver (in this case all were mothers). While the components of the intervention varied by level, common targets for all levels included improved handwashing and infection control (including clean delivery environment at the health center level), recognition of newborn danger signs, and prompt referral when these were present. The at-home intervention also included rapid assessment for newborn danger signs and measurement of body temperature. More detail on the intervention is presented below in Table 1.

The initial exploratory outcome was all-cause mortality by study group, but due to concerns over the ability to accurately assess this outcome given the small population size, this outcome was not ultimately calculated as part of the formal analysis.

Primary Outcomes

- % of mothers/caregivers who know at least three danger signs of newborn illness
- % VHSG who know six danger signs of newborn illness
- % of caregivers who seek care from an appropriate facility
- Time between onset of suspected danger signs and referral to appropriate facility
- Improved infection control behavior among HC staff

Secondary Outcomes

- % of newborns visited by VHSG within the first day of life
- % of newborns visited by VHSG after the first day of life
- % VHSG who can deliver hygiene messages
- % of mothers who received messages on hygiene from HC staff
- % of mothers who received messages on hygiene from VHSG
- % of mothers who received messages on care-seeking from VHSG
- % HC staff who know six danger signs of newborn illness
- % HC staff who recall hygiene messages

### 2.3. Sample Size

The initial exploratory outcome of newborn mortality and procedures for stepped wedge design for cluster-randomized trials were used to generate a target total sample size of 1761 newborns based on 80% power and at the 5% significance level to detect a 20% reduction, with a baseline neonatal mortality of 39 deaths/1000 live births in target provinces and an intraclass correlation coefficient of 0.0007256. Other intervention phase outcomes with higher baseline rates than all-cause mortality were hypothesized to demonstrate 20% or greater improved outcome in the intervention group (e.g., incidence of newborns with specific danger signs, proportion of newborns with danger signs referred to an appropriate facility), but the lack of reliable baseline data on these did not allow us to use them for power calculations.

### 2.4. Participants

Inclusion criteria for community-level participants were women in their last trimester of pregnancy who were healthy and able to participate in the intervention activities (home visits and data collection), able to adhere to the study protocol, aged greater than 18 years old, and who gave birth at a catchment HC. Any participants for whom the index pregnancy did not result in a live birth were excluded due to difficulties in assessing stillbirth status. We excluded births with known congenital malformation or in cases where there was an inability to provide informed consent or adhere to the study protocol. Informed consent was obtained by reading information sheets to the participants and formally asking their consent to participate in the study by signature or thumbprint. Following consent, the study staff administered the enrollment questionnaire. After the birth in their catchment HC, NICCI study staff collected data from participants on the 14th and 28th day of the newborn’s life.

The health center-level intervention training (at cluster level) included the HC chief (a physician), all midwives, and any other professional staff involved with mother and newborn care, as well as janitorial staff where these were present.

The cluster unit was an individual health center including health center catchment area villages and all beneficiaries of those catchment areas who would be eligible for maternity services at the time of the study. Over a period of 16 months, as assigned by randomization, the clusters transitioned from control to intervention condition. After a one-half day training session on registration of pregnant women, trained VHSGs began registering women reporting to be in their last trimester of pregnancy. Health Centers with more than 20 deliveries per month were eligible to participate, and all VHSGs supervised by participating health centers were included.

### 2.5. Randomization

**Figure 1:**
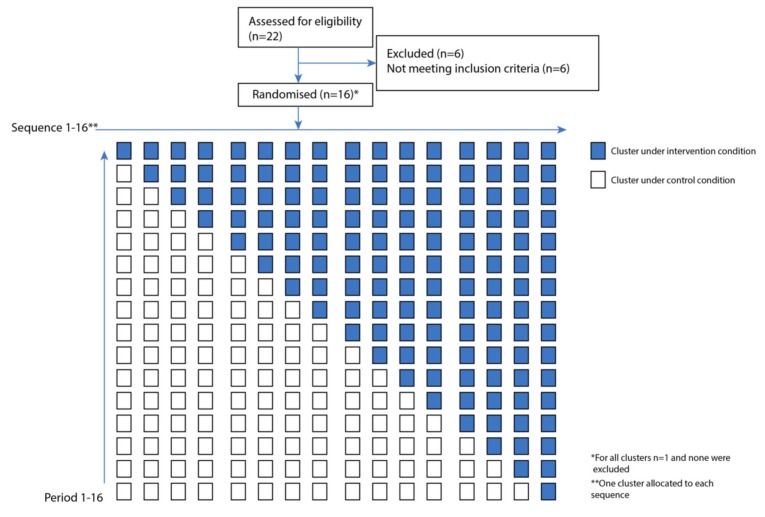
CONSORT Stepped Wedge Cluster Randomized Controlled Trial Flow Chart. Flow chart.

**Figure 2:**
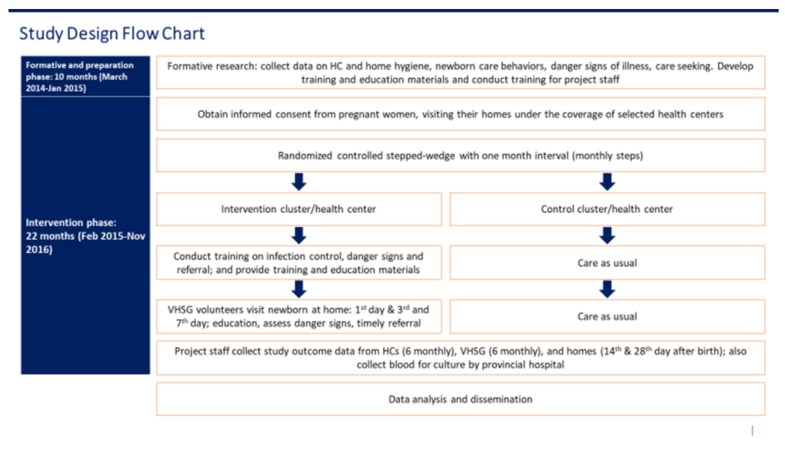
Study timeline and participant flow. HC: health center.

Two figures are included below to illustrate the study design and flow of participants. Figure 1 presents the cluster assignment and stepped wedge design, per the CONSORT checklist extension format for reporting of stepped-wedge cluster-randomized trials [23], while Figure 2 presents the timeline, including randomization of cluster start, monthly step wise flow, and detailed information on participants. Participants were blinded to group assignment.

### 2.6. Implementation, Care Coordination and Data Collection

Ministry of Health Maternal Child Health (MCH) Focal Persons at the operational district (OD) and provincial levels, who are appointed by the government health sector to provide close supervision of maternal and child health services, provided 2 day training sessions to HC staff as well as the VHSGs under the HC catchment area in order to begin the intervention.

Topics for training of intervention cluster health staff included handwashing, infection prevention and control for delivery and postdelivery rooms, sterilization procedures, newborn danger signs, breastfeeding counseling, and essential newborn care. Teaching methods included a brief explanation of procedures followed by participatory and interactive modules, including group discussion, role play, and demonstration and practice, as well as the opportunity to discuss constraints and problem solving for quality care for newborns, and infection control. The action plan then developed was used for monitoring progress every 6 months for process evaluation.

VHSG volunteers were trained on handwashing, newborn assessment for danger signs, referral process, and essential newborn care counseling (including breastfeeding, bathing, cord care, skin-to-skin contact, and care for low-birth-weight baby). During the training, where each session had 20–30 participants, VHSGs performed role-playing simulations on how to conduct home visits. Following training, intervention VHSG received tools developed as part of a formative study [9]. The NICCI trial team distributed one educational flipchart, one thermometer for each VHSG, and one hand phone set per village for VHSG to contact HC staff. The study team conducted reviews of VHSG volunteers every two months.

The health center registry was used to record deliveries, following which staff contacted VHSGs by phone on discharge, with contact information and report of newborn status. The study team developed a list of relevant HC staff with their phone number and provided the list to relevant VHSG under each HC. NICCI provided a USD 2 phone card per village per month to VHSGs in all intervention villages.

Participants enrolled in the study who met all inclusion criteria were interviewed by the study team three times: (1) during the last trimester of her pregnancy (at baseline enrollment), (2) the 14th day after delivery, and (3) on the 28th day after delivery (NICCI allowed the flexibility of interviewing mothers two days before or after the 14th/28th day). The study team also collected other data, such as reports of newborn sepsis from newborns who were hospitalized in Takeo Provincial Hospital, and data from verbal autopsy of newborn deaths. Due to concerns over the ability to accurately assess newborn mortality given the small population size, the trial team ultimately did not seek to assess newborn mortality rate or detect differences between deaths among intervention and control groups.

### 2.7. Statistical Methods and Data Analysis

Descriptive statistics were generated to define the population’s baseline characteristics for comparison between intervention and control groups. The differences between the two groups were compared using Pearson’s Chi-squared *P*-values for categorical variables, Student’s t-test for normally distributed continuous variables and Wilcoxon rank-sum tests for skewed continuous variables. Date are presented as mean with interquartile range (IQR) or numbers with percentages. All statistical analyses were completed using an intent-to-treat approach, i.e., all the eligible individuals’ assignments were based on delivery date and delivery facility.

Outcomes were divided into three levels: health facility level, community level, and patient level. For health facility and community level, data from each interview was applied into a logistic generalized estimating equation (GEE) model with repeated measurement (assessed every 6 months) [24]. For primary outcomes at patient level, both bivariate models and multivariable models based on GEE models adjusted for clustering (health care) were applied to (i) percentage of mothers who know at least three danger signs; (ii) percentage of families who seek care from an appropriate facility; (iii) decreased time between onset of suspected danger signs and referral to appropriate facility; and (iv) improved infection prevention behaviors by family. The bivariate model accessed intervention effect only, and the multivariable model also included significant potential covariates. The logit link function was used for dichotomous variables, and identify link function was used for dimensional variables. An unstructured covariance matrix was used for GEE models to prevent incorrect assumptions of the covariance structure and allow flexibility. Exponents of the coefficients of independent variables in the models were selected “step-wisely” to correspond with the study design, where potentially significant (*p*-value < 0.1) variables were entered first, then the least significant variables removed step by step. All retained exposure variables were significant in the final multivariable model (*p*-value < 0.05) at the last step, e.g., in the multivariable model for percentage of mothers who know at least three danger signs [25]. Results of comparison of intervention and control groups were interpreted by odds ratio (OR), and standard errors for the coefficients were used to estimate *p*-values and associated 95% confidence intervals (95% CI) for the OR. All statistical tests were interpreted in a two-tailed fashion, and the analyses were performed using SAS Version 9.4 (SAS Institute Inc., Cary, NC, USA) [26].

The ethics approval was granted through Tulane University IRB approval number 534777-4 and also through the Cambodian National Ethics Committee for Health Research IRB number NECHR397.

## 3. Results

In the course of the intervention, 113 HC staff and 504 VHSGs were trained to perform their tasks. From February 2015 to November 2016, 2597 enrolled mothers were interviewed on the 14th day after delivery, 2483 mothers were interviewed on the 28th day after delivery. The final trial data analysis was based on 2494 complete records of participants meeting all inclusion criteria (including 11 participants whose newborns died between day 14 and 28 of the study). Data were also collected from 565 individual VHSG volunteers and 92 Heath Center staff.

Table 2 illustrates that the participants in control and intervention clusters had similar baseline sociodemographic characteristics, and no statistically significant differences were observed.

Table 3 shows the effect of the intervention on improving knowledge and behavior at the health center (HC) level. HC staff in the intervention group had vastly more likelihood of handwashing at the recommended key moments with soap or disinfectant in provision of care when compared with the control group, as measured by self-report. HC staff who knew six danger signs in the intervention group reached 98% compared with only 15% of those in control group. Similarly 95% of intervention staff recalled at least three hygiene messages compared with 54% in the control group.

Table 4 shows the effect on knowledge and performance of VHSG (through home visit). Notably 60.58% of VHSGs in the intervention group knew at least six danger signs compared with only 2.11% of VHSGs in the control group (the mean knowledge score of VHSG regarding hygiene was 4.00 in the intervention group compared with 2.60 in the control group).

Our analysis also indicated that 24% of newborns in the intervention group received more than one visit by VHSG compared with 16% in the control group. Within the first day of life and after the first day of life, 25.67% and 26.50% of the newborns in the intervention group received a visit from VHSG, respectively, compared with 16.31% and 13.82% in the control group.

Table 5 shows the positive impact of the intervention on mother/caretaker knowledge. At 14 days after delivery, women in the intervention group were more likely to know at least three danger signs compared with women in the control group (OR = 2.35, *p* = 0.0104). Effects were not evident on care-seeking behavior, e.g., seeking care from appropriate health facilities, as measured by time between noticing danger signs and time the newborn was referred or handwashing score.

Table 5 also illustrates effects on the performance of the HC staff and VHSG in providing counseling, where 12.89% of mothers in the intervention group received messages on hygiene from VHSG volunteers compared with 1.5% in the control group, and 35.01% of mothers in the intervention group received messages on care-seeking from VHSG volunteers compared with 5.35% in the control group.

Eleven newborn deaths of infants born alive could be verified during the one-month follow-up period. Six of these occurred in the intervention group (*n* = 1691, mortality rate 0.35%) and 5 in the control group (*n* = 803, mortality rate 0.62%). This difference was not statistically significant (Mantel–Haenszel Chi-Square *p* = 0.34).

## 4. Discussion 

Overall, the intervention had a positive impact on community health worker and mother knowledge of newborn danger signs, as well as HC staff knowledge for counseling, newborn care practices, and recognition of danger signs. Self-reported behavior of HC staff on handwashing also improved significantly. While knowledge indicators were greatly improved for HC staff, VHSG, and mothers of newborns, there was no significant effect of the intervention on self-reported handwashing among families, on care-seeking, and referral. Rates of VHSG home visits to mothers of newborns were statistically different and improved in the intervention group, but the overall rates were still less than 25%, indicating the difficulty of improvement in this outcome. Training and regular supervision may motivate VHSG volunteers to better perform in-home visits and in providing health message to the mothers [18].

Considering all three target groups (HC staff, VHSG, and mothers) the intervention was associated with improved knowledge of danger signs and hygiene, and improved performance by health professionals in meeting key indictors (infection prevention practices, home visits). However health care-seeking behaviors of mothers were less impacted. There was a nonsignificant trend toward improved referral time in the intervention group, but the time for referral was less than 6 h for only about 17% of newborns. The delay in referral time for sick newborns underscores the transportation challenges in rural Cambodia, where public transportation is lacking and mothers are usually dependent on informal networks of friends for emergency transportation, often by motorcycle.

In one study, delay in treatment-seeking outside home was associated with 81% of newborn deaths [27]. The current trial did not have effect on early referral of newborns; however study data not reported in the tables above indicated that having a means of transportation, e.g., motorcycle, car, did increase likelihood of bringing a sick infant to an appropriate facility (*p* = 0.004). Undoubtedly, means of transportation of the household is likely an important determining factor.

Enabling factors for improved water, hygiene, and sanitation at the household level may be related to lack of effect of the NICCI trial on certain behaviors among community participants. Water, sanitation, and hygiene are among the important components for improving newborn mortality [28]. Research has illustrated the potential for reduction in newborn illness, including newborn sepsis deaths, following clean birth and clean postnatal care practices [28,29]. Training on WASH (Water Sanitation Hygiene) and Infection Prevention Control (IPC), including appropriate practices in cleaning the birthing environment, for both health care providers and ward cleaners, represents an important opportunity for quality improvement. Acceptance of these practices by local populations has been shown to be important in the success of WASH-based interventions [30], and simultaneous community-based antenatal care education on possible danger signs is key in this process. Previous studies on community health worker effectiveness have noted the importance of WASH practices in maternal and neonatal health, especially at the level of the health center [31,32]. However, it is not possible to determine the effect on outcomes of training on knowledge beyond 28 days of delivery without cohort or longitudinal studies. The effects beyond the newborn period were outside the scope of the current study because the intended focus was on the illness during the vulnerable newborn period.

Regarding how this study advances research in the field, this is the first study in the country to investigate newborn health through an intervention trial, and the first linked study in Cambodia to address neonatal health at the level of the facility, village health worker, and household. Strengths of the trial include the stepped-wedge cluster-randomized design, which allows for robust statistical evaluation in resource-poor settings. A linked approach [20] has been advocated as crucial because often prenatal and postnatal interventions are not well integrated into maternal and newborn health services [33]. Healthy facility infection prevention control can have a strong impact on maternal and neonatal survival, as contrasted with a recent study in Tanzania [34]. Limitations included self-report of handwashing/infection prevention, challenges in assessing fidelity, potential bias from any missed participants in clusters (e.g., women who did not eventually deliver at their assigned health center), and inability to isolate the most effective components of intervention. Since blinding was not possible for health care staff, there is a limitation due to the reliability of self-report, and as missed data add significant bias, this limits external validity of our results. Trends suggestive of more rapid referral practices and decreased infant mortality in the intervention group would have benefited from a larger sample size.

## 5. Conclusions

The data and results from the trial illustrated that training using experiential activities, focused on skills and practice, with regular supervision, improved the knowledge and behavior of HC staff and VHSG volunteers, as well as mothers’ knowledge of newborn danger signs. Infection prevention control, home visits by community-based volunteers, and maternal counseling are known to be closely associated with newborn survival and health. Further improvement in handwashing behavior among families will need to involve broader stakeholders in the community, including strengthening community engagement and resources for improving water and sanitation infrastructure. As the VHSG staff are volunteers, further efforts to incentivize higher rates of home visits need to be evaluated.

## Figures and Tables

**Table 1:** Summary of Intervention Components.

Health Center Level: Staff	Infection Prevention Control refresher training—including handwashing, clean cord care, clean linens and birthing mats Clean delivery environment Newborn danger signs and prompt referral Use of data collection forms and recording
Community Level: VHSG	Hygiene and handwashing practices Newborn danger signs, quick assessment of infant, and taking axillary temperature Prompt, appropriate referral
Household: Mothers/caregivers	Hygiene and handwashing practices Breastfeeding Newborn danger signs Prompt, appropriate referral
Visits: Within 24 h of birth	Counseling of mothers/caregivers Hygiene practices/handwashing Essential newborn care (reinforce breastfeeding) Newborn danger signs Prompt, appropriate referral Assessment of newborn danger signs and rapid assessment of infant (axillary temperature)
Visits: Day 3	Counseling of mothers/caregivers Hygiene practices Essential newborn care (reinforce breastfeeding) Newborn danger signs Prompt, appropriate referral Assessment of newborn danger signs and rapid assessment of infant (axillary temperature)
Visits: Day 7	Counseling of mothers/caregivers Hygiene practices Essential newborn care (reinforce breastfeeding) Newborn danger signs Prompt, appropriate referral Assessment of newborn danger signs and rapid assessment of infant (axillary temperature)

**Table 2:** Demographic characteristics of participants.

Characteristics of Interest	Control	Intervention
	*N* = 803	*N* = 1691
*Demographic characteristics*		
• Age, mean (IQR)	26.7 (23.0–30.0)	26.7 (23.0–30.0)
• No previous pregnancies, N(%)	314 (39.1)	655 (38.7)
• Previous pregnancies, N(%)		
▪ Three or more previous	36 (7.4)	78 (7.5)
• Education, N(%)		
▪ No schooling	47 (5.9)	82 (4.9)
▪ Primary	325 (40.5)	667 (39.5)
▪ Lower and upper	427 (53.1)	894 (53.0)
▪ Higher than upper	4 (0.5)	47 (2.8)
• Assets, household ownership, N(%)		
▪ Car	20 (2.5)	41 (2.4)
▪ Motorcycle	661 (82.3)	1441 (83.4)
• Housing, N(%)		
▪ Sole owner	334 (41.6)	726 (42.9)
▪ Living at relative's house	458 (57.0)	939 (55.5)
▪ Other (joint homeowner, renting, other)	11 (1.5)	26 (1.5)
• Farm land property, N(%)		
▪ Own farm land	477 (59.4)	1020 (60.4)
▪ Other (parent's land, rented land, no farm land)	326 (40.7)	670 (39.6)
• Occupation, N(%)		
▪ Farmer	316 (39.4)	419 (24.8)
▪ Factory worker	183 (22.8)	684 (40.5)
▪ Housewife	208 (26.0)	354 (20.9)
▪ Other (vendor/seller, civil servant, business woman, other)	96 (12.0)	234 (13.9)
*Other characteristics*		
• Household with electricity, N(%)	694 (86.4)	1340 (79.2)
• Latrine, N(%)		
▪ Any latrine	536 (66.8)	1187 (70.2)
▪ No latrine	267 (33.3)	504 (29.8)
• Water for drinking boiled, N (%)	595 (74.1)	1298 (76.8)
• Handwashing practice		
▪ Not wash hands	2 (0.3)	3 (0.2)
▪ Water only	44 (5.5)	121 (7.2)
▪ Water with soap	590 (73.5)	989 (58.5)
▪ Other (water with detergent, ash, other)	311 (38.8)	1123 (66.4)
• Family has a “PoorID card”	174 (21.7)	356 (21.1)

**Table 3:** Health facility outcome measures.

Outcome	Results		OR (CI)	*p*-Value
	Intervention	Control		
Infection prevention and control behavior among HC staff (measured by proportion of appropriate handwashing with soap or disinfectant at key points in provision of care to mothers and newborns)	52.88%	6.71%	15.60 (7.73–31.47)	<0.0001
HC staff knowledge of danger signs (measured by % of HC staff who know at least six danger signs)	98.45%	14.77%	35.91 (19.31–66.78)	<0.0001
HC staff ability to recall hygiene messages (as measured by % of HC staff who recall at least three hygiene messages)	95.29%	54.36%	16.98	<0.0001

**Table 4:** VHSG training and newborn home visits.

Outcome	Results		OR (CI)	*p*-value
	Intervention	Control		
VHSG knowledge of danger signs (as measured by % of VHSG who know six danger signs)	60.58%	2.11%	71.44 (41.55–122.87)	<0.0001
Percent of newborns visited at least *once* by VHSG on or before day 7 of life	24.25%	15.94%	1.69	<0.001
Percent of newborns visited at least *twice* by VHSG on or before day 7 of life	24.24%	16.04%	1.77	<0.001
VHSG knowledge of hygiene (as measured by % of VHSG who can deliver hygiene messages)	67.75%	18.10%	9.51	<0.0001

**Table 5:** Outcomes at community level.

Outcome	Results		OR (CI)	*p*-Value
	Intervention	Control		
Mothers’ knowledge of danger signs (% of mothers who know at least three danger signs)	54.9%	30.5%	2.35 (1.22–4.52)	0.0104
Care-seeking (% of families who sought care from appropriate facility)	80.20% (Frequency only)	81.82% (Frequency only)	*	*
Referral (time between onset of suspected danger signs and referral to appropriate facility: <6 h)	17.28%	36.84%	0.45 (0.19–1.06)	0.0688
Infection prevention (as measured by proportion of mothers’ reporting handwashing with soap at key events)	57.78%	37.36%	1.62 (0.81–3.22)	0.1697
Counseling by VHSG (% of mothers who reported receipt of messages on the following topics): Essential newborn care Handwashing Care-seeking	36.15% 11.1.% 35.01%	5.53% 15.1% 5.35%	10.66 0.70 9.52	0.0005 0.5342 <0.0001

* Regression model could not be applied due to zero in a cell.
